# Altered Mental Status and Respiratory Failure in an 11-Year-Old Female

**DOI:** 10.7759/cureus.15164

**Published:** 2021-05-22

**Authors:** Emily R Ribeiro, Floyd Livingston, Madelyn Kahana, Robert Smith

**Affiliations:** 1 Pediatrics, Nemours Children's Hospital, Orlando, USA; 2 Pulmonology, Nemours Children's Hospital, Orlando, USA; 3 Pediatric Critical Care Medicine, Nemours Children's Hospital, Orlando, USA

**Keywords:** phox2b, late onset congenital central hypoventilation syndrome, hypoventilation, tracheostomy, central congenital hypoventilation

## Abstract

Congenital central hypoventilation syndrome (CCHS) is a rare disorder that results in profound hypoventilation that is most prominent during periods of sleep. Caused by a genetic mutation in the *PHOX2B* gene, CCHS typically presents in the newborn period with symptoms of hypoventilation. However, there is a subset of patients with the same genetic mutation who present much later in life, which is termed late-onset congenital central hypoventilation syndrome (LO-CCHS). The reason for its late presentation is unclear but is often dramatic. Given its rarity, the diagnosis can be difficult to establish but can be accomplished by using a systematic approach. Here, we present a case of LO-CCHS in an 11-year-old female who presented with respiratory failure and altered mental status.

## Introduction

In German mythology, there is a story of a water nymph named Ondine, who falls in love with a mortal. When she discovers that he has been unfaithful to her, she curses him to stop breathing should he ever go to sleep. The myth of “Ondine’s curse” has made its way into modern medicine by way of a disorder called congenital central hypoventilation syndrome (CCHS). This disorder, caused by a mutation in the *PHOX2B* gene, is characterized by the failure of the autonomic control of respiration. This ultimately leads to hypoxia and hypercapnia, particularly while the patient is asleep [1].

Typically, patients present in early infancy with cyanosis and hypoventilation. However, there is a subset of patients who present much later in life, and these patients ultimately are diagnosed with late-onset congenital central hypoventilation syndrome (LO-CCHS). It is currently unclear why there is a difference between the presentation of CCHS and LO-CCHS, though variations in *PHOX2B* mutations, variable copy numbers, variable expressivity, and environmental cofactors have been proposed mechanisms [1-4]. Regardless of when patients present, they ultimately require the same diagnostic testing, treatment, and management. Here, we present a case of LO-CCHS diagnosed in an 11-year-old female who had presented with respiratory failure and altered mental status.

## Case presentation

An 11-year-old female with a history of seizure disorder controlled on Trileptal, learning disability, attention deficit hyperactivity disorder, and pica presented with altered mental status and vomiting. She was ill with fever, cough, nausea, and anorexia for two weeks prior to presentation and had completed a five-day course of azithromycin to treat atypical pneumonia. On the morning of presentation, she had multiple episodes of emesis and an inability to tolerate oral intake, which prompted evaluation in the emergency department.

In the emergency department, she was found to be lethargic and hypoxic with oxygen saturation in the 70s on room air. Vital signs showed a temperature of 36.8°C, heart rate of 100 beats per minute, blood pressure of 120/71 mmHg, and respiratory rate of 19 breaths per minute. She was placed on high-flow nasal cannula 20 liters per minute and 0.5 FiO_2_. Physical examination showed a lethargic child in no acute distress. Neurologic examination revealed cranial nerves II-XII intact, and normal speech, motor, and sensation examinations. Respiratory effort was normal, and there were end-expiratory rales bilaterally. She was mildly tachycardic with regular rhythm and normal distal perfusion. Abdominal examination was benign without hepatosplenomegaly.

**Figure 1:**
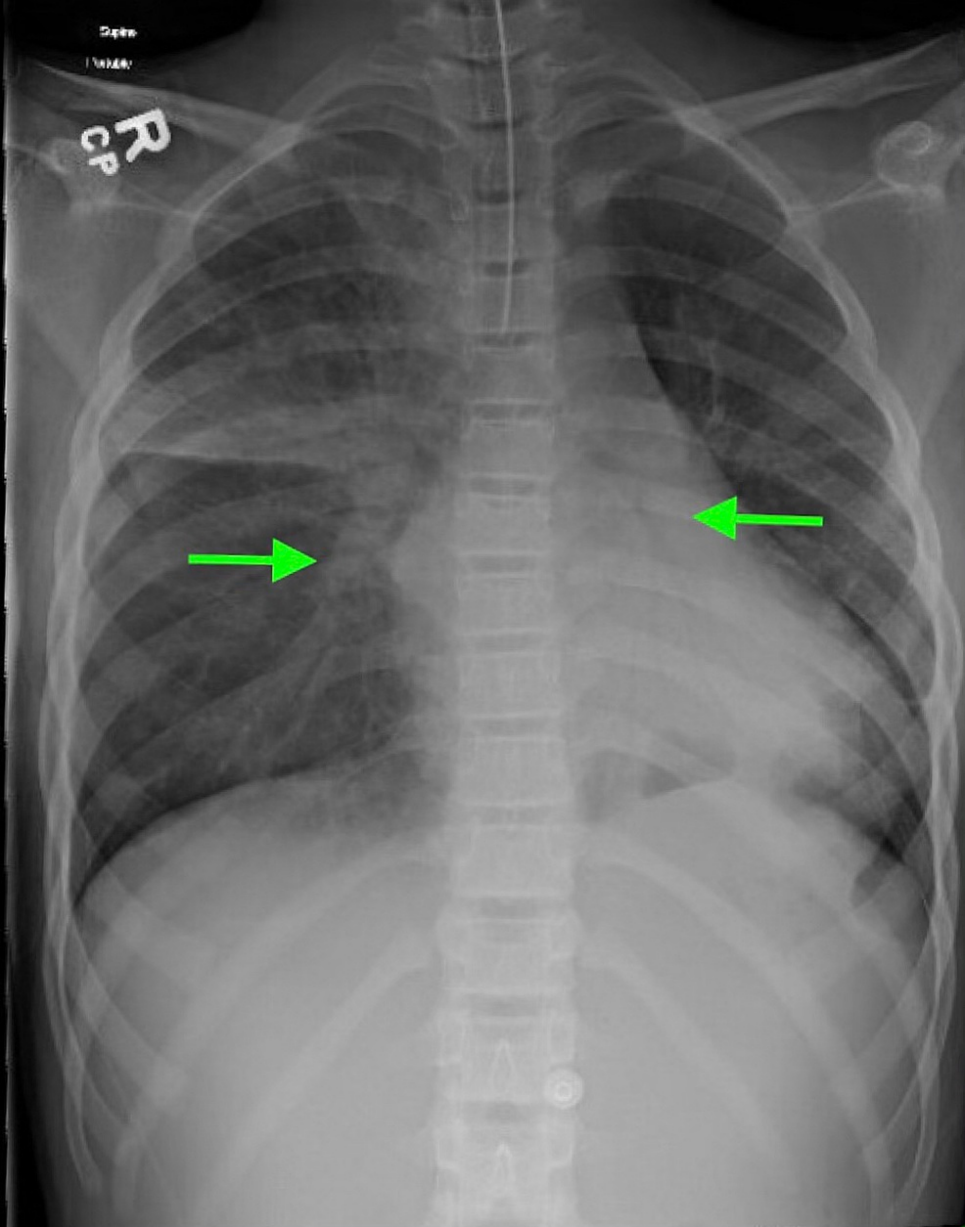
Chest X-ray of the patient on admission showing bilateral perihilar opacities (arrows)

Initial laboratory evaluation showed venous blood gas of pH 7.39, pCO_2_ of 65 mmHg (8.64 kPa), and HCO_3_ of 40 mEq/L (40 mmol/L). Chemistry was significant for sodium of 130 mEq/L (130 mmol/L), chloride of 87 mEq/L (87 mmol/L), bicarbonate of 37 mEq/L (37 mmol/L), blood urea nitrogen (BUN) of 28 mg/dL (10 mmol/L), and creatinine of 0.57 mg/dL (43.46 µmol/L). Liver enzymes were increased with aspartate aminotransferase (AST) of 1,396 U/L (23.31 µkat/L) and alanine aminotransferase (ALT) of 1,003 U/L (16.75 µkat/L). Urine drug screen was negative. Chest X-ray, as read by the radiologist, revealed bilateral perihilar opacities with no focal infiltrate (Figure 1). On admission to the pediatric intensive care unit, she was escalated to BiPAP (bilevel positive airway pressure) support, but due to progressive alteration in mentation, she was orally intubated for acute hypoxic and hypercapnic respiratory failure. Shortly after intubation, she was awake, alert, and requested a quick extubation.

After extubation, repeat physical and neurologic examinations were nonfocal with normal cranial nerve function. However, it was noted that her carbon dioxide levels continued to rise. During the day, she could be coached to take deep breaths to lower her carbon dioxide level. At night, her carbon dioxide levels rose to 96 mmHg (12.8 kPa), which could only be controlled with noninvasive ventilation. Multiple studies including drug screen, thyroid studies, lead levels, hepatitis screen, cerebrospinal fluid cell counts and cultures, and urine electrolytes were sent and normal. Encephalopathy panel revealed N-type calcium channel receptor, for which the patient was started on intravenous immunoglobulin. Head imaging with CT and MRI were normal. On further history taking, it was revealed she had significant intellectual impairment and tremendous daytime somnolence with failing school performance. In combination with the fact that most laboratory studies were negative or unrevealing and that she had no respiratory drive in response to hypercarbia, genetic studies for CCHS were sent on day 2 of hospitalization and resulted positive for heterozygous c.765_779dup *PHOX2B* gene variant on day 22 of hospitalization.

After the diagnosis of LO-CCHS was made, a multi-disciplinary team including pulmonology, neurology, otolaryngology, social work, and child life met with the family to discuss treatment options. After discussing options with the family and patient, she had a tracheostomy placed and was discharged on invasive mechanical ventilation only during the night time and at times of rest. She had improvement in lab values following tracheostomy placement, as venous blood gas revealed pH of 7.40, pCO_2_ of 43 mmHg (5.7 kPa), and HCO_3_ of 26.4 mEq/L (26.4 mmol/L). Chemistry results were as follows: sodium of 135 mEq/L (135 mmol/L), chloride of 98 mEq/L (98 mmol/L), bicarbonate of 33 mEq/L (33 mmol/L), BUN of 10 mg/dL (3.57 mmol/L), and creatinine of 0.2 mg/dL (15.25 µmol/L). She was discharged home after 43 days of hospitalization.

At home with tracheostomy ventilation at night time, she has done remarkably well with significant improvement in mental status and excessive daytime tiredness. Her end-tidal carbon dioxide has been maintained between 35 to 45 mmHg (4.7 to 5.9 kPa) with normalization of her acid-base balance. She has returned full-time to school and has accelerated to the top 25% of her class within eight months of treatment.

## Discussion

Given the nature of her presentation, the differential for this patient was quite broad. She had presented with a mixed acid-base status, of which the primary disorder was unclear. Patients with a primary metabolic alkalosis may be asymptomatic or complain of symptoms related to the underlying etiology. The differential for metabolic alkalosis includes drug use, mineralocorticoid excess, and renal disorders, all of which were tested upon admission for this patient and were negative. However, rarely does a primary metabolic alkalosis cause such a dramatic presentation of respiratory failure seen in this patient. Therefore, a compensated primary respiratory acidosis was more likely.

The differential for respiratory failure is long and includes anything that can involve the respiratory pathway, from the central nervous system to the lungs. Starting from the central nervous system, there are numerous disease processes that can decrease central respiratory drive in the brainstem such as sedatives, encephalitis, brainstem lesions, or hypothyroidism. Testing for our patient revealed N-type calcium channel receptor on encephalitic panel. Treatment was initiated, but it was ultimately determined that the testing was of no clinical significance as there are no reports of N-type calcium receptor encephalitis leading to hypoventilation, and this patient subsequently tested positive for a *PHOX2B* mutation. Head imaging studies and lab tests were otherwise negative.

Respiratory centers in the brain respond to input from both central chemoreceptors (which sense changes in pH and carbon dioxide) and peripheral chemoreceptors (which primarily sense changes in oxygen), meaning that disorders in detecting changes in oxygen, carbon dioxide, or pH can lead to altered ventilation [2]. The brain then provides a neuronal drive to respiratory muscles, and therefore neuromuscular disorders such as a spinal injury, amyotrophic lateral sclerosis, Guillain-Barre syndrome, myasthenia gravis, or thoracic cage disorders can also lead to this presentation. Neuromuscular disease was not high on the differential for this patient given her nonfocal neurologic examination while awake, though testing was sent prior to establishment of diagnosis for this patient. The differential for a primary lung issue is expansive but can broadly be thought of as diseases of ventilation/perfusion mismatch. There are a variety of other pathologies, including toxins, fever, or upper airway disorders, that can cause issues throughout the respiratory center. Our patient exhibited no obvious cause of ventilation/perfusion mismatch.

Given that this patient had no obvious source of respiratory failure, genetic disorders were considered early in this patient’s course, as genetic disorders can affect the respiratory system anywhere along the aforementioned respiratory pathway. One such genetic mutation is CCHS, a rare disorder characterized by the failure of the autonomic control of respiration leading to hypoxia and hypercapnia, particularly while the patient is asleep [1]. In CCHS, patients are unable to sense changes in carbon dioxide, leading to a lack of regulation of ventilation in response to changing carbon dioxide levels. This is most apparent during sleep, particularly non-rapid eye movement sleep, when breathing is almost entirely dependent on metabolic control [2]. Most often, patients present in the newborn period with cyanosis and hypoventilation during sleep or with severe central sleep apnea.

However, there is a small percentage of patients who are diagnosed later in life and present with unexplained apnea, respiratory failure, or seizures [2]. These patients may only present after an event such as sedation, anesthesia, or respiratory infection [3]. Given their late onset, they are appropriately diagnosed with LO-CCHS. Regardless of when patients present, they ultimately require the same diagnostic testing, treatment, and management.

For both CCHS and LO-CCHS, disease-causing mutation is on the *PHOX2B* gene [2]. *PHOX2B* is thought to be a master gene for the neuronal network for the autonomic nervous system [5]. Given its widespread expression, there are multiple non-respiratory symptoms associated with *PHOX2B* mutations, and therefore CCHS and LO-CCHS, including cardiac conduction abnormalities, impaired thermoregulation, diminished pupillary light reflexes, esophageal dysmotility, and learning and developmental delays [2,3]. Additionally, around 20% of patients also have Hirschsprung’s disease, and tumors of neural crest origin are not uncommon [3].

In around 90% of cases, the genetic mutation is a polyalanine repeat in exon 3 [2,4]. Our patient was heterozygous for the c.765_779dup gene variant, which results in an in-frame duplication (p.Ala256_Ala260dup) and therefore expansion of the polyalanine strand. The mutation is considered pathogenic for CCHS [6].

It is hypothesized that the degree of polyalanine expansion is related to severity of symptoms, which could explain why some patients present with hypoventilation symptoms later in life [1,3,4]. Indeed, our patient exhibited a relatively small number of repeats, which could explain her late presentation. Other mutations in *PHOX2B* have been implicated [4], and mutations in other genes have also been proposed to have syndromes similar to CCHS [3].

Ultimately, patients will need genetic testing for definitive diagnosis [4]. For LO-CCHS, testing is often only sent when patients begin exhibiting symptoms of hypoventilation. However, if a patient has a history of associated symptoms such as unusually long breath-holding, constipation, and neurocognitive delays, the diagnosis can be suspected and genetic testing can be sent much earlier [4].

While awaiting confirmatory testing, which took around 20 days for our patient, other potential causes of hypoventilation, such as cardiac disease, muscle weakness, or a primary lung disease, should be ruled out [4]. Cardiac evaluation is typically done with an electrocardiogram to evaluate for sinus pauses and right ventricular hypertrophy, and potentially an echocardiogram for further evaluation of right ventricular hypertrophy and pulmonary hypertension [4]. Our patient underwent an echocardiogram and Holter monitoring, which were both normal. Neurologic evaluation includes brain imaging with MRI or CT for brain or brainstem lesions [4], and head CT and MRI were normal for our patient. Lung disease can be ruled out with chest imaging as well as by polysomnogram. Chest X-ray for our patient was not concerning for focal processes. Polysomnogram was attempted early on, which was concerning for sleep apnea but was not diagnostic of LO-CCHS. Finally, physiologic evaluation with blood gases both while awake and asleep can be suggestive of LO-CCHS [4], which is what led to concern for this patient.

The gold standard of treatment for these patients is chronic invasive ventilation [2,4]. Multiple options exist, including positive pressure ventilators via tracheostomy, BiPAP, negative-pressure ventilators, or diaphragm pacing [2,4]. Noninvasive ventilation can be considered but is not favored [2]. Currently, there are no pharmacologic interventions [3].

Following definitive diagnosis, patients with LO-CCHS will require further screening for other autonomic nervous system dysfunction, including barium enema or rectal biopsy, to evaluate for Hirschsprung’s disease, cardiac rhythm monitoring to evaluate for arrhythmias, and chest and abdomen imaging for neural crest tumors [2]. Follow-up for these patients is crucial, and these screenings should be conducted yearly [4]. With adequate treatment and follow-up, many patients are living well into adulthood and have a good quality of life [4].

Finally, given the genetic basis of this disorder, family members also require genetic testing. *PHOX2B* is inherited in an autosomal-dominant, incompletely penetrant manner, with variable expressivity [1,2,4]. This means that though it is a dominant gene, not every person will exhibit symptoms nor will they all exhibit the same symptoms. It is thought that mild phenotypes may go unnoticed and that some mutations may require an environmental cofactor to elicit symptoms [3,4]. Essentially, all family members in the pedigree must be tested [4]. Even if testing is negative, the parents of a child with a *PHOX2B* mutation should still have prenatal testing for subsequent pregnancies in case there is a germline mutation [4].

## Conclusions

LO-CCHS is a rare disorder caused by a mutation in the PHOX2B gene that is characterized by dysregulated control of respiration and is most pronounced during sleep. Most patients with this mutation present in infancy with respiratory failure, and therefore diagnosis may not be considered for older individuals. The differential for respiratory failure is broad, but diagnosis of LO-CCHS can and should be suspected if there is a history of long breath-holding spells, constipation, and neurocognitive delays. Definitive diagnosis requires genetic testing. Treatment for LO-CCHS is with chronic non-invasive or invasive mechanical ventilation to maintain normal carbon dioxide levels and oxygenation. Management of LO-CCHS includes screening for Hirschsprung’s disease, cardiac rhythm abnormalities, and neural crest tumors. Family members require genetic testing given its autosomal-dominant, incompletely penetrant inheritance.
